# Production of (2R, 3R)-2,3-butanediol using engineered *Pichia pastoris*: strain construction, characterization and fermentation

**DOI:** 10.1186/s13068-018-1031-1

**Published:** 2018-02-12

**Authors:** Zhiliang Yang, Zisheng Zhang

**Affiliations:** Department of Chemical and Biological Engineering, University of Ottawa, 161 Louis Pasteur Private, Ottawa, ON K1N 6N5 Canada

**Keywords:** *Pichia pastoris*, 2,3-Butanediol, Metabolic engineering, Medium optimization

## Abstract

**Background:**

2,3-butanediol (2,3-BD) is a bulk platform chemical with various potential applications such as aviation fuel. 2,3-BD has three optical isomers: (2R, 3R)-, (2S, 3S)- and meso-2,3-BD. Optically pure 2,3-BD is a crucial precursor for the chiral synthesis and it can also be used as anti-freeze agent due to its low freezing point. 2,3-BD has been produced in both native and non-native hosts. Several pathogenic bacteria were reported to produce 2,3-BD in mixture of its optical isomers including *Klebsiella pneumoniae* and *Klebsiella oxytoca*. Engineered hosts based on episomal plasmid expression such as *Escherichia coli*, *Saccharomyces cerevisiae* and *Bacillus subtilis* are not ideal for industrial fermentation due to plasmid instability.

**Results:**

*Pichia pastoris* is generally regarded as safe and a well-established host for high-level heterologous protein production. To produce pure (2R, 3R)-2,3-BD enantiomer, we developed a *P. pastoris* strain by introducing a synthetic pathway. The *als*S and *als*D genes from *B. subtilis* were codon-optimized and synthesized. The BDH1 gene from *S. cerevisiae* was cloned. These three pathway genes were integrated into the genome of *P. pastoris* and expressed under the control of GAP promoter. Production of (2R, 3R)-2,3-BD was achieved using glucose as feedstock. The optical purity of (2R, 3R)-2,3-BD was more than 99%. The titer of (2R, 3R)-2,3-BD reached 12 g/L with 40 g/L glucose as carbon source in shake flask fermentation. The fermentation conditions including pH, agitation speeds and aeration rates were optimized in batch cultivations. The highest titer of (2R, 3R)-2,3-BD achieved in fed-batch fermentation using YPD media was 45 g/L. The titer of 2,3-BD was enhanced to 74.5 g/L through statistical medium optimization.

**Conclusions:**

The potential of engineering *P. pastoris* into a microbial cell factory for biofuel production was evaluated in this work using (2R, 3R)-2,3-BD as an example. Engineered *P. pastoris* could be a promising workhorse for the production of optically pure (2R, 3R)-2,3-BD.

**Electronic supplementary material:**

The online version of this article (10.1186/s13068-018-1031-1) contains supplementary material, which is available to authorized users.

## Background

Driven by depleting fossil fuels and growing environmental concern, production of bulk chemicals from renewable sources via biosynthesis is becoming increasingly desirable [1]. 2,3-Butanediol (2,3-BD) is a crucial platform chemical with enormous applications. It can be converted to other platform chemicals such as methyl ethyl ketone, gamma-butyrolactone and 1,3-butadiene [2]. 2,3-BD has three stereoisomers: (2R, 3R)-, (2S, 3S)- and meso-2,3-BD [3]. Enantiopure isomers of 2,3-BD could serve as the precursor for asymmetric synthesis and synthetic rubber. It is also used as an anti-freeze agent due to its low freezing point. 2,3-BD is also a good alternative liquid fuel owing to its high energy capacity. Biosynthesis of 2,3-BD has been reported in many microorganisms. Native producers belonging to *Enterobacteriaceae* family such as *Klebsiella pneumoniae*, *Klebsiella oxytoca* and *Enterobacter aerogenes* were able to produce high titer of 2,3-BD in mixture of its optical isomers [1]. Despite the high titer achieved in those hosts, chiral purity of 2,3-BD was not satisfactory. Moreover, the use of risk group 2 microorganisms projects a safety concern for industrial fermentation. Production of 2,3-BD via fermentation of GRAS (generally regarded as safe) bacteria has also been reported. Several *Bacillus* species including *B. subtilis* [4], *B. amyloliquefaciens* [5] and *B. licheniformis* [6] are effective producers of 2,3-BD. Jurchescu and coworkers reported 144.7 g/L 2,3-BD production using B. *licheniformis* DSM 8785, a risk group 1 bacterium [7]. *Paenibacillus polymyxa* ZJ9 was reported to produce 36.92 g/L (2R, 3R)-2,3-BD with 98% purity from Jerusalem artichoke tubers under optimized conditions [8]. Engineered hosts based on plasmid expression of heterologous pathway genes from the aforementioned microorganisms were reported. The use of non-native hosts such as *Escherichia coli* [9, 10] and *Saccharomyces cerevisiae* [11–13] offers several advantages due to their well-established genetics, higher metabolic rate, simpler process control and lack of competing pathways. Lian and coworkers reported the production of 100 g/L 2,3-BD in *S. cerevisiae* using glucose and galactose as carbon source [14].

The methylotrophic yeast *Pichia pastoris* has been recognized as an excellent host for heterologous protein production. Its success as a workhorse for recombinant protein production was attributed to the ability to perform post-translational modifications, high secretion efficiency of proteins and simple nutrition requirements [15]. It is also ideal for industrial fermentation due to the GRAS status. *P. pastoris* can be grown to high cell density on defined medium using crude glycerol, a by-product of biodiesel production, as carbon source [16]. *P. pastoris* has been engineered into microbial cell factory for the production of biofuel in recent years. *P. pastoris* was used as whole cell catalysts to produce biodiesel through surface display of lipases [17, 18]. Other bio-products produced by engineered *P. pastoris* included lycopene [19], β-carotene [20], xanthophylls [21], (+)-nootkatone [22], dammarenediol-II [23] and lactic acid [24]. In this work, we aim to produce chiral pure (2R, 3R)-2,3-BD in *P. pastoris* using metabolic and process engineering strategies. The potential of *P. pastoris* as a promising host for 2,3-BD production was demonstrated for the first time.

## Methods

### Strains, plasmids and reagents

*Escherichia coli* XL1-Blue was used for plasmid cloning and propagation. *P. pastoris* X33 was used as host for metabolic engineering. *E. coli* was grown in low-salt LB broth (1% peptone, 0.5% yeast extract and 0.5% sodium chloride) supplemented with 25 µg/mL of Zeocin where appropriate. *P. pastoris* was cultivated in YPD media (1% yeast extract, 2% peptone and 2% dextrose) with 100 µg/mL of Zeocin. YPDS plates consisting of 1% yeast extract, 2% peptone, 2% dextrose, 18% sorbitol and 2% agar were used for yeast transformant screening. Plasmid pGAPZαA (Invitrogen, USA) was used for gene cloning. Chemicals of analytical grade and restriction enzymes used in this work were purchased from Sigma-Aldrich (USA) or Fisher scientific (Canada). DNA primers were ordered from Invitrogen (USA) and nucleotide sequences of primers are listed in Table 1. *E. coli* or yeast genomic DNA was purified using Gentra Puregene Yeast/Bac. Kit (Qiagen, Germany). Polymerase chain reaction (PCR) was performed using KAPA HiFi Hotstart Readymix PCR Kit (Kapa Biosystems, USA).

**Table 1:** Primers used in this study

Primer name	Sequence (5′–3′)
AlsS-f	CGGGGTACCATGTTGACTAAGGCTACTAAGGAACAAA
AlsS-r	TCCCCGCGGTTACAAAGCTTTAGTCTTCATCAAC
AlsD-f	CGGAATTCAAAATGAAGAGAGAGTCCAACATCCAAG
AlsD-r	CGGGGTACCTTATTCTGGTGATCCCTCGGTTGTT
BDH1-f	CGGAATTCAAAATGAGAGCTTTGGCATATTTCAAGA
BDH1-r	CGGGGTACCTTACTTCATTTCACCGTGATTGTTA
udhA-f	CGGGGTACCAAAATGCCACATTCCTACGATTACGATG
udhA-r	TGCTCTAGATTAAAACAGGCGGTTTAAACCGTTT
HIS4-f	GAAGATCTATGACATTTCCCTTGCTACCTGC
HIS4-r	CGGGATCCTTAAATAAGTCCCAGTTTCTCCATACG

Restriction sites are underlined

### DNA manipulation

To clone the 2,3-BD biosynthesis genes under the control of GAP promoter, pGAPZαA was digested with *Bsp*119I and *Eco*RI to remove the α-signal sequence. The large fragment was gel-purified and blunted by DNA blunting enzyme (CloneJET PCR cloning kit, Thermo scientific, USA). The treated fragment was self-ligated to result in a plasmid pGAPZ which is used for gene expression in this work. DNA sequences of biosynthesis pathway genes *B. subtilis* 168 *alsS* and *alsD* were retrieved from National Center for Biotechnology Information (NCBI). Sequences of *alsS* and *alsD* were codon-optimized according to the *P. pastoris* codon usage to achieve better expression. The optimized genes of *alsS* and *alsD* were synthesized by Genscript (USA) and inserted into vector pUC57, resulting in pUC57-AlsS and pUC57-AlsD, respectively. *alsS* gene was amplified from pUC57-AlsS using AlsS-f and AlsS-r. The PCR product was digested with *Kpn*I and *Sac*II and ligated into pGAPZ digested with the same enzymes to obtain pGAPZ-AlsS. *alsD* was cloned into pGAPZ to result in pGAPZ-AlsD with the same manner using *Eco*RI and *Kpn*I. 2,3-Butanediol dehydrogenase gene BDH1 was amplified from *S. cerevisiae* genomic DNA using BDH1-f and BDH1-r and cloned into pGAPZ between *Eco*RI and *Kpn*I to obtain pGAPZ-BDH1. pGAPZ-AlsD was digested with *Bam*HI and *Bgl*II to obtain a fragment containing P_GAP_-AlsD-tAOX1. pGAPZ-AlsS was linearized with *Bam*HI and ligated with P_GAP_-AlsD-tAOX1 to result in plasmid pGAPZ-SD. BDH1 was cloned into pGAPZ-SD in the same manner to result in pGAPZ-SDB. To integrate the pathway genes into HIS4 locus of the *P. pastoris* genome, HIS4 gene was PCR amplified from *P. pastoris* X33 genomic DNA using primers HIS4-f and HIS4-r. HIS4 was cloned into pGAPZ-SD and pGAPZ-SDB to result in pGAPZ-SDH and pGAPZ-SDBH, respectively. *E. coli udhA* gene was amplified from *E. coli* XL1-blue genomic DNA using primers udhA-f and udhA-r. *udhA* was first cloned into pGAPZ to result in pGAPZ-udhA and then inserted into pGAPZ-SD to obtain pGAPZ-SDU. HIS4 was inserted into pGAPZ-SDU, resulting in pGAPZ-SDUH. All plasmid constructs were confirmed by PCR and sequencing.

### Yeast transformation and screening

*Pichia pastoris* X33 was made competent and transformed with various plasmids (Table 2) using an electroporator (Eppendorf, Canada) according to the manufacturer’s instructions. Briefly, 5 µg of pGAPZ-SDH, pGAPZ-SDBH, pGAPZ-SDUH and pGAPZ-SDBUH were linearized with *Nhe*I and transformed into competent *P. pastoris* X33 cells to obtain strains X33-SD, X33-SDB and X33-SDU, respectively. Yeast transformants were screened on YPDS plates by incubating at 30 °C for 2–3 days. Yeast colonies were picked up and grown in YPD media. Genomic DNA was extracted and used as PCR templates for the confirmation of pathway gene integration.

**Table 2:** Plasmids and strains used in this study

Strains or plasmids	Description	Source
*E. coli* XL1-blue	recA1 endA1 gyrA96 thi-1 hsdR17 supE44 relA1 lac [F´ proAB lacIq Z∆M15 Tn10 (Tetr)]	Lab stock
*P. pastoris* strains		
X33	Wilde type	Lab stock
X33-SD	*P. pastoris* X33 harbouring codon-optimized AlsS and AlsD gene	This study
X33-SDB	*P. pastoris* X33 harbouring codon-optimized AlsS and AlsD and *S. cerevisiae* BDH1 gene	This study
X33-SDU	*P. pastoris* X33 harbouring codon-optimized AlsS and AlsD and *E. coli* udhA gene	This study
Plasmids		
pGAPZαA	GAP promoter, α-signal, Zeocin resistance	Invitrogen
pGAPZ	GAP promoter, Zeocin resistance	This study
pGAPZ-AlsS	pGAPZ harbouring AlsS gene	This study
pGAPZ-AlsD	pGAPZ harbouring AlsD gene	This study
pGAPZ-BDH1	pGAPZ harbouring BDH1 gene	This study
pGAPZ-udhA	pGAPZ harbouring udhA gene	This study
pGAPZ-SD	pGAPZ harbouring AlsS and AlsD genes	This study
pGAPZ-SDB	pGAPZ harbouring AlsS, AlsD and BDH1 genes	This study
pGAPZ-SDU	pGAPZ harbouring AlsS, AlsD and udhA genes	This study
pGAPZ-SDH	pGAPZ harbouring AlsS, AlsD and HIS4 genes	This study
pGAPZ-SDBH	pGAPZ harbouring AlsS, AlsD, BDH1 and HIS4 genes	This study
pGAPZ-SDUH	pGAPZ harbouring AlsS, AlsD, udhA and HIS4 genes	This study

### Shake flask cultivation

Single yeast colony was inoculated into 50-mL Falcon tube containing 10 mL YPD media and grown overnight. Shake flask cultivation was performed by inoculating 1 mL of overnight culture into 100 mL YP media (1% yeast extract, 2% peptone) containing various concentrations of glucose in a 500-mL shake flask. Aliquots were taken every 4–6 h. Samples were centrifuged at 13,000 rpm for 5 min. Supernatant was filtered through 0.22-µm filter and used for further analysis.

### Bioreactor setup

Batch and fed-batch fermentations were performed in 5-L bioreactors (Bioflo320, Eppendorf, Canada) equipped with two Rushton impellers. Overnight yeast culture (5% v/v) was inoculated into the fermentation media. Bioreactors were kept at 30 °C. The cultivation was performed at the optimal condition with pH 5.0 (± 0.02), agitation speed of 300 rpm and aeration rate of 0.5 vvm unless otherwise specified. The pH was maintained constant by adding 30% ammonia hydroxide through on-line pH monitoring. Feeding solution was fed to the bioreactor once the glucose was depleted. Glucose feed rate was adjusted between 0.2 and 0.8 mL/min to maintain a low concentration of glucose to avoid substrate inhibition.

### Effect of cultivation conditions on titer of 2,3-BD in YPD

Batch fermentation of *P. pastoris* strain was performed in 5-L bioreactors (Bioflo320, Eppendorf, Canada) containing 3 L YPD media. Bioreactor inoculum was prepared by streaking a single colony from the plate and growing in a 500-mL shake flask containing 150 mL YPD media overnight (30 °C and 250 rpm). Effect of fermentation conditions was investigated by varying one parameter (pH, agitation speed and aeration rate) at a time. Other cultivation conditions were kept unchanged when one parameter was varied.

### Statistical medium optimization

Basal salt medium (BSM) consisting of per liter: 42.9 g KH_2_PO_4_, 14.33 g K_2_SO_4_, 5.17 g (NH_4_)_2_SO_4_, 5.7 g MgSO_4_·7H_2_O, 0.6 g CaSO_4_·2H_2_O and 4 mL PTM1 was used for medium optimization. Trace metal solution PTM1 consists of per liter: 6 g CuSO_4_·5H_2_O, 0.08 g NaI, 3 g MnSO_4_·H_2_O, 0.2 g Na_2_MoO_4_·2H_2_O, 0.02 g HBO_3_, 0.5 g CoCl_2_, 20 g ZnCl_2_, 65 g FeSO_4_·7H_2_O, 0.2 g biotin and 5 mL H_2_SO_4_. Minitab 15 was used to generate Plackett–Burman and Box–Behenken design matrix and perform statistical analysis. Shake flask cultivation was performed to screen the significant factors affecting the titer of 2,3-BD. The responses (2,3-BD titer) were used to fit a first-order model. Box–Behnken design was employed to determine the optimal level of the three significant factors. The 2,3-BD titers obtained in shake flask cultivation were used to fit a quadratic model. Fed-batch cultivation using the optimized medium recipe was performed at 30 °C, pH 5.0, 300 rpm and 0.5 vvm.

### Analytical methods

Biomass was monitored by measuring optical density at 600 nm (OD_600_) with Ultraspec 60 (UK). Glucose concentration was measured using YSI2900 Bio-analyzer (Mandel, Canada) installed with a glucose membrane. 2,3-BD isomers were differentiated using gas chromatography (GC, Agilent 6850 series, Santa Clara, CA, USA) equipped with flame ionized detector (FID). For GC-FID analysis, yeast cultures were centrifuged at 13,000 rpm for 5 min. The supernatant was extracted with equal volume of ethyl acetate. Organic phase was dehydrated with sodium sulphate before GC analysis. The capillary GC column (Supelco Astec CHIRALDEX™ B-PM, 35 m × 0.25 mm × 0.12 µm) was used in this study. Helium was used as the carrier gas with a flow rate of 2 mL/min. The injector and detector were kept at 250 °C. Injection volume was 1 µL. Oven temperature program [25] was as follows: 1.5 min at 50 °C, programmed to increase up to 160 °C at a rate of 8.8 °C/min and hold for 5 min at 160 °C. Extracellular metabolites were determined by high-performance liquid chromatography (HPLC, Agilent 1200 series, Santa Clara, CA, USA) equipped with a Shodex Sugar SH1011 column (8 mm ID × 300 mm, 6 µm; Showa Denko, Tokyo, Japan). The column was maintained at 60 °C and eluted with 5 mM sulphuric acid at a flow rate of 0.6 mL/min. Acetoin was detected with variable wavelength detector at 214 nm. Other metabolites were detected with refractive index detector.

### Statistical analysis

Student test was used to compare mean values using Origin 8.0. For medium optimization, statistical analysis was carried out using Minitab 15. Difference with confidence level of 95% (*P* < 0.05) was considered statistically significant.

## Results

### Construction of *P. pastoris* strains for the production of (2R, 3R)-2,3-BD

**Fig. 1:**
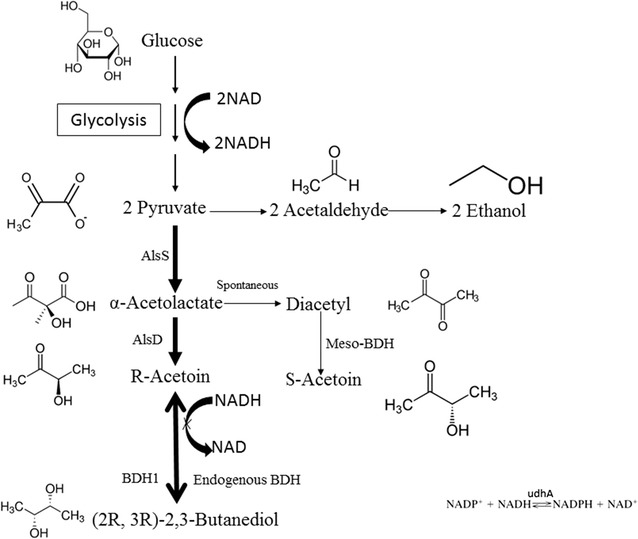
Engineered 2,3-BD synthesis pathway in this work. The 2,3-BD synthesis pathway was indicated by bold arrows

**Fig. 2:**
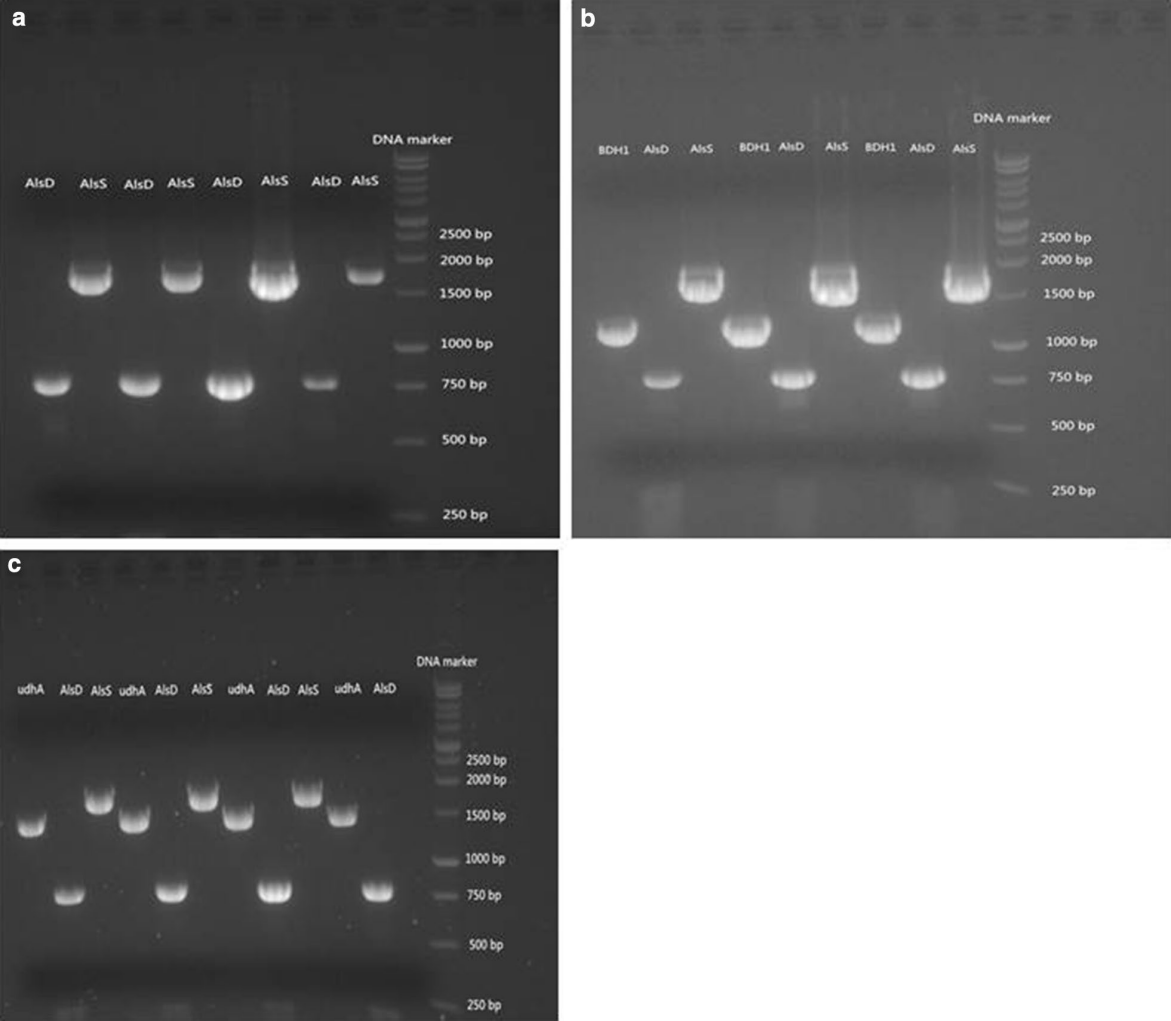
PCR confirmation of constructed strains. **a** PCR verification of X33-SD. Genomic DNA of four yeast colonies was extracted and used as templates for PCR. **b** PCR verification of X33-SDB. Genomic DNA of three yeast colonies was extracted and used as templates for PCR. **c** PCR verification of X33-SDU. Genomic DNA of four yeast colonies was extracted and used as templates for PCR. DNA bands and DNA markers are indicated in the pictures

**Fig. 3:**
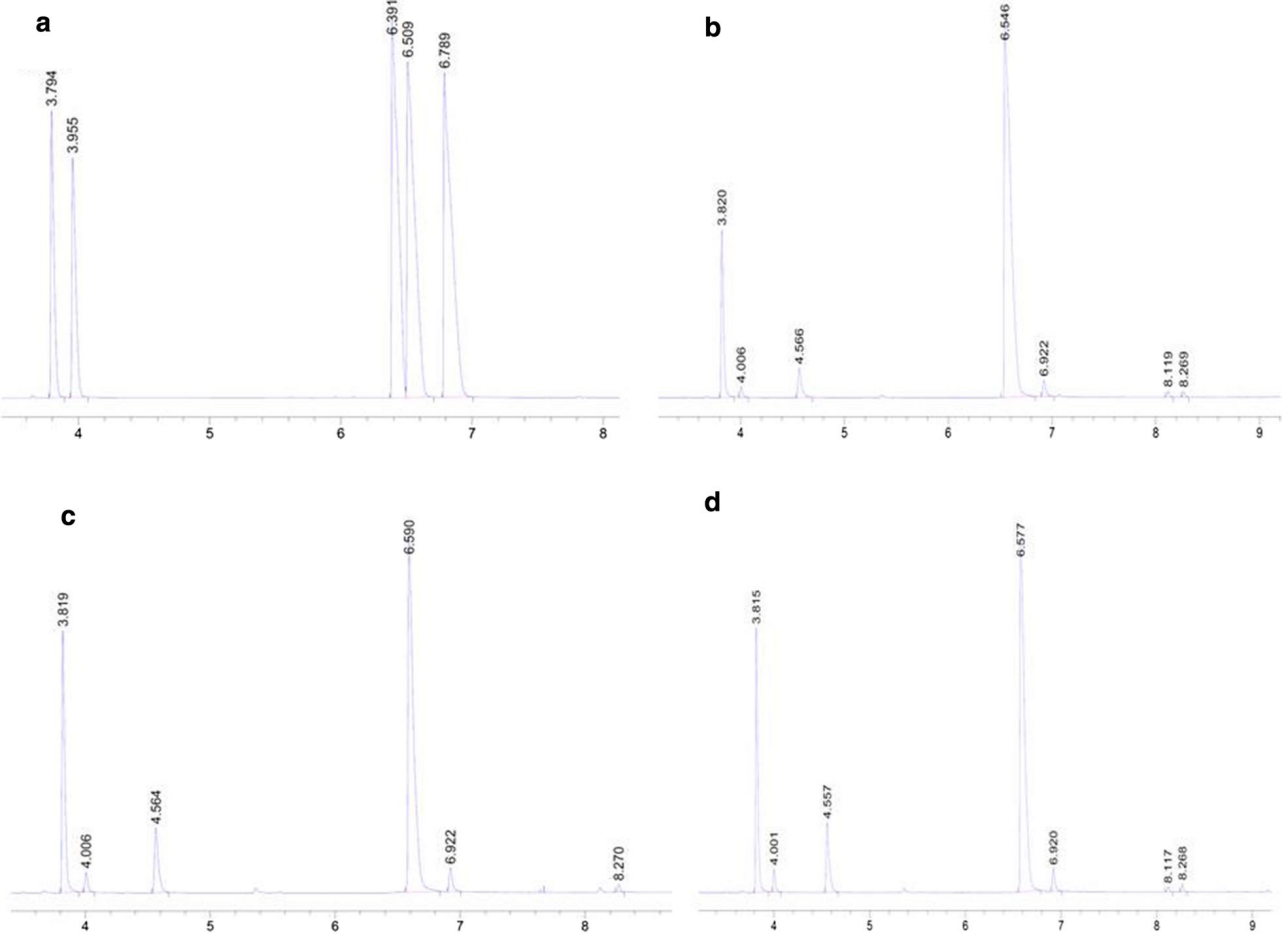
GC profiles of metabolites in different strains. **a** GC profile of standards. Retention times: 3S-acetoin, 3.8 min; 3R-actoin, 4.0 min; (2S, 3S)-2,3-BD, 6.4 min; (2R, 3R)-2,3-BD, 6.5 min; meso-2,3-BD, 6.8 min. **b** GC profile of extract of culture of X33-SD. **c** GC profile of extract of culture of X33-SDB. **d** GC profile of extract of culture of X33-SDU

*Pichia pastoris* is an industrially relevant host for recombinant protein production and has not been reported to produce 2,3-BD from glucose up to date. Previous study described the conversion of acetoin to 2,3-BD using *P. pastoris* through NADH (nicotinamide adenine dinucleotide) regeneration [26]. 2,3-BD is produced from pyruvate via three enzymatic steps (Fig. 1). To introduce a synthetic route for 2,3-BD production in *P. pastoris*, *B. subtilis* α-acetolactate synthase AlsS, *B. subtilis* α-acetolactate decarboxylase AlsD and *S. cerevisiae* (2R, 3R)-2,3-BD dehydrogenase BDH1 were selected to direct the carbon flux towards 2,3-BD synthesis. Constitutive promoter P_GAP_ is commonly used for protein expression in *P. pastoris* due to its high-level constitutive expression and growth-associated product formation [27]. The three pathway genes were cloned under the control of P_GAP_ for constitutive expression. Codon optimization of *alsS* and *alsD* was performed to achieve better expression in *P. pastoris* [28]. The pathway genes were assembled into a single plasmid for chromosome integration at the HIS4 locus of *Pichia* genome. As shown in Fig. 2, *alsS*, *alsD*, *udhA* and BDH1 were cloned into vector pGAPZ and successfully integrated into *P. pastoris* genome via homologous recombination. The constructed strains were grown in YPD media to verify 2,3-BD production. The optical purity of 2,3-BD is determined by the stereo-specificity of 2,3-BD dehydrogenase (Fig. 1). As shown in Fig. 3, recombinant *P. pastoris* strains X33-SD, X33-SDB and X33-SDU were able to produce enantiopure (2R, 3R)-2,3-BD using glucose as feedstock. Optical purity was determined to be over 99%. Trace amount of meso-2,3-BD was produced. (2S, 3S)-2,3-BD was not detected, indicating the stereo-specificity of endogenous 2,3-butanediol dehydrogenase (2,3-BDH) is specific for (2R, 3R)-2,3-BD synthesis.

### Screening of different strains for high 2,3-BD production

**Fig. 4:**
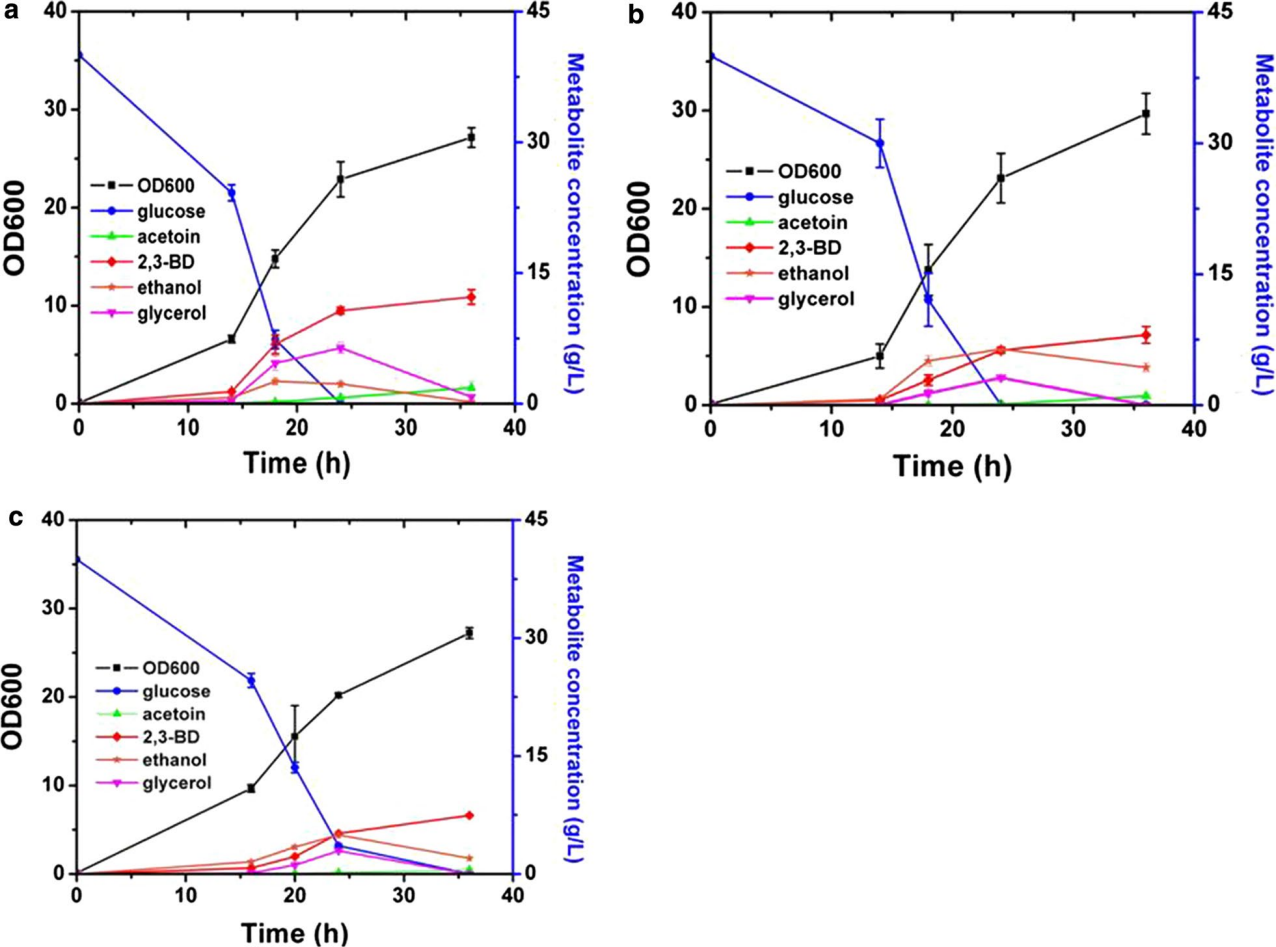
Screen of recombinant strains in shake flasks. **a** Time course of X33-SD in 500-mL shake flask containing 100 mL YP media with 40 g/L glucose. **b** Time course of X33-SDB in 500-mL shake flask containing 100 mL YP media with 40 g/L glucose. **c** Time course of X33-SDU in 500-mL shake flask containing 100 mL YP media with 40 g/L glucose. Cultivation last for 36 h. Error bars indicate the standard deviation of three replicate experiments

Three strains constructed in this work were cultivated to evaluate their potential for 2,3-BD production in shake flasks containing 40 g/L glucose (Fig. 4). Yeast cultures were grown for 36 h and aliquots were analysed. As shown in Fig. 4, glucose was depleted at 24 h. Major by-products detected in the broth were glycerol, acetoin and ethanol. No acetic acid was detected in all three strains. Cell growth was not significantly affected by the 2,3-BD synthetic pathway (Table 3). Strain X33-SD, X33-SDB and X33-SDU produced 2,3-BD with a titer of 12.2 ± 0.84, 8.04 ± 0.95 and 7.44 ± 0.19 g/L, respectively. Surprisingly, overexpression of *S. cerevisiae* BDH1 was not beneficial to boost 2,3-BD production. This result is contradictory with previous study [26]. The *E. coli udhA* gene for NADH regeneration from NADPH (nicotinamide adenine dinucleotide phosphate) did not improve 2,3-BD titer as well. The highest acetoin titer was observed in strain X33-SD, reaching 1.84 ± 0.76 g/L. Acetoin consists mainly of S-acetoin with minor R-acetoin detected based on the GC analysis (Fig. 3). Despite *P. pastoris* is generally recognized as Crabtree effect-negative, ethanol is produced as a by-product under anaerobic condition. Wild-type *P. pastoris* X33 produced 15 g/L ethanol after 36 h cultivation (data not shown). The highest ethanol titer of X33-SD, X33-SDB and X33-SDU was detected at 24 h, with 2.28 ± 0.36 g/L, 6.43 ± 0.07 g/L and 4.96 ± 0.07 g/L, respectively. Strain X33-SD produced 0.21 ± 0.19 g/L ethanol after 36 h, in contrast with 4.3 ± 0.52 g/L and 1.99 ± 0.01 g/L obtained with X33-SDB and X33-SDU, respectively. Ethanol titer decreased at the end of cultivation because it could be consumed after glucose depletion. Compared with wild-type X33, the heterologous 2,3-BD synthetic pathway effectively redirected the carbon flux from ethanol formation towards 2,3-BD synthesis.

**Table 3:** Comparison of metabolite production of three *P. pastoris* strains

Strains	X33-SD	X33-SDB	X33-SDU
OD_600_	27.15 ± 1^α^	29.68 ± 2.08^α^	27.23 ± 0.62^α^
2,3-BD (g/L)	12.2 ± 0.84^α^	8.04 ± 0.95^β^	7.44 ± 0.19^β^
Acetoin (g/L)	1.84 ± 0.76^α^	1.03 ± 0.09^α^	0.54 ± 0.07^β^
Glycerol (g/L)	6.42 ± 0.62^α^	3.14 ± 0.28^β^	2.94 ± 0.23^β^
Ethanol (g/L)	2.28 ± 0.36^α^	6.43 ± 0.08^β^	4.96 ± 0.07^γ^

Cultivation of three strains was carried out in 500-mL shake flasks containing 100 mL YP media with 40 g/L glucose. Cultivation last for 36 h. Data indicates average values of triplicate experiments with standard deviation. Values with different Greek symbols between columns were significantly different (*P* < 0.05). ND indicates not detected

Glycerol was produced as a major by-product as a channel for the regeneration of NAD^+^ in engineered *S. cerevisiae* for the production of 2,3-BD [29]. Notably, 6.42 ± 0.62, 3.14 ± 0.28 and 2.94 ± 0.23 g/L glycerol was accumulated in strain X33-SD, X33-SDB and X33-SDU, respectively. Production of glycerol in *P. pastoris* has never been reported in the literature. Analysis of the *P. pastoris* genome sequence could offer a possible mechanism for glycerol formation. Two putative glycerol-3-phosphate dehydrogenases (GPDs) were present in *P. pastoris* genome. Putative GPD (PAS_ch2_2_0111) shares 57% amino acid similarity with GPD2 in *S. cerevisiae*. Further investigation is imperative to verify the enzyme activity of the putative GPDs in *P. pastoris*. Strain X33-SD is the most promising strain in terms of highest 2,3-BD production and least ethanol accumulation and is thus used for further study (Table 3).

### Effect of glucose concentration

**Fig. 5:**
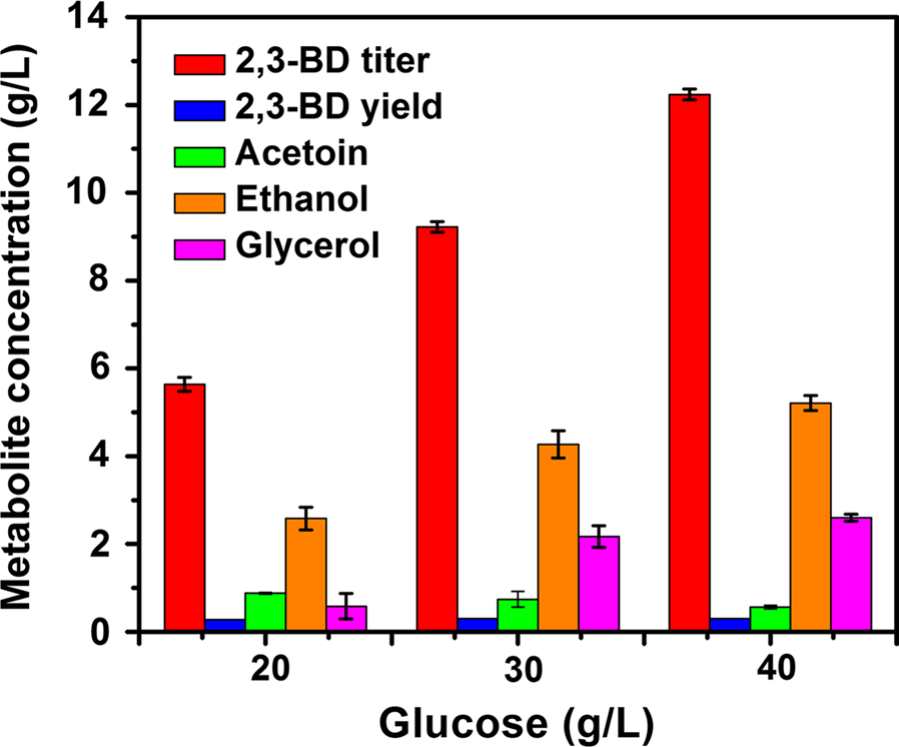
Effect of glucose concentration. Strain X33-SD was cultured in 500-mL shake flask containing 100 mL YP media containing 20, 30 and 40 g/L glucose. Error bars indicate standard deviation of three replicate experiments

Yeast strain X33-SD was cultivated in shake flasks with YPD medium containing different initial concentrations of glucose. As shown in Fig. 5, higher titer of 2,3-BD was obtained at higher glucose concentration, reaching 5.64 ± 0.16, 9.22 ± 0.12 and 12.24 ± 0.15 g/L with glucose concentration of 20, 30 and 40 g/L, respectively. Higher concentrations of ethanol and glycerol were also observed with higher glucose concentrations. Acetoin was detected below 1 g/L for all three glucose concentrations. The 2,3-BD yield on glucose remained about 0.3 g/g for the three glucose concentrations tested, which is 60% of the theoretical yield (0.5 g/g).

### Effect of fermentation conditions on 2,3-BD production

#### Effect of agitation

**Fig. 6:**
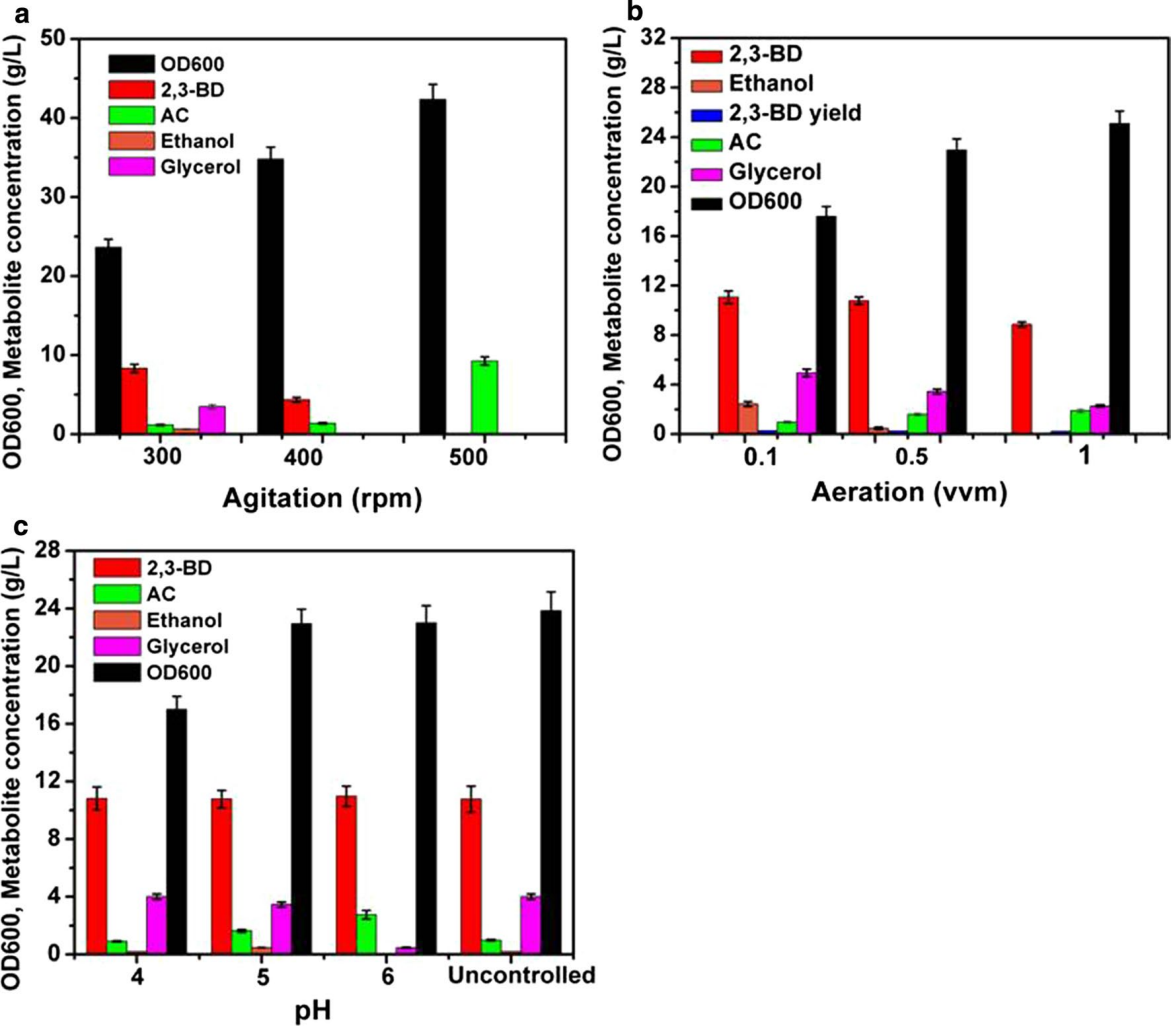
Effect of cultivation conditions on 2,3-BD titer. Strain X33-SD was cultivated in 3 L YPD media in 5-L Bioflo320 reactors. Glucose concentration was 40 g/L. Error bars indicate standard deviation of duplicate experiments. **a** Effect of agitation. **b** Effect of aeration. **c** Effect of pH

Bioreactor cultivations of strain X33-SD were performed to investigate its potential for large-scale production of (2R, 3R)-2,3-BD. Fermentation conditions such as agitation speeds, aeration rates and pH were optimized under controlled conditions. Effect of agitation is illustrated in Fig. 6a. Three agitation speeds were examined: 300, 400 and 500 rpm with an aeration rate of 1.5 vvm. It was found that metabolite profile was closely related to agitation speed. Production of 2,3-BD was detected under 300 and 400 rpm but not detected under 500 rpm. The highest titer of 2,3-BD was achieved at 300 rpm, reaching 8.34 ± 0.62 g/L. Acetoin was the major product at 500 rpm with a titer of 9.27 ± 0.81 g/L. Glycerol and ethanol were accumulated only at 300 rpm. The highest biomass concentration was obtained at 500 rpm with an OD_600_ of 42. 35 ± 2.61, compared with 23.65 ± 1.12 and 34.8 ± 1.89 at 300 rpm and 400 rpm, respectively. It was reported that lower dissolved oxygen (DO) level favours the reduction of acetoin to 2,3-BD [1]. DO is normally controlled by cascading agitation and aeration where agitation is more prominent to DO control. Optimization of agitation speed for improved production of 2,3-BD has been reported in previous studies. Xu and coworkers optimized the agitation speed within the range of 200 to 500 rpm using engineered *E. coli* and found that 400 rpm was optimal in terms of high biomass accumulation and 2,3-BD production [30]. Two-stage agitation control strategy has been implemented to cultivate *K. oxytoca* to achieve a balance between biomass production and 2,3-BD synthesis. Agitation was maintained at 300 rpm in the first 15 h for biomass growth and lowered to 200 rpm for the production of 2,3-BD [31]. Two-stage agitation improved the titer by 6.2% compared with constant speed of 200 rpm. The agitation-associated metabolite profile suggests that strain X33-SD could be used to produce acetoin under higher agitation speed.

#### Effect of aeration

Aeration is another major factor to determine DO level. We tested three aeration rates: 0.1, 0.5 and 1 vvm with agitation speed of 300 rpm. As was shown in Fig. 6b, higher aeration resulted in higher biomass production. OD_600_ reached 25.1 ± 1.2 at 1 vvm compared with 17.6 ± 0.9 at 0.1 vvm. The titer of 2,3-BD obtained at 0.1 vvm was slightly higher than that at 0.5 vvm, reaching 11.07 ± 0.81 and 10.78 ± 0.65 g/L, respectively. Only 8.87 ± 0.43 g/L 2,3-BD was detected at 1 vvm. Acetoin was produced at 0.97 ± 0.03, 1.6 ± 0.07 and 1.89 ± 0.15 g/L at 0.1, 0.5 and 1 vvm, respectively. The highest glycerol concentration of 4.95 ± 0.56 g/L was observed at 0.1 vvm. Ethanol was not detected at 1 vvm whilst 0.46 and 2.43 ± 0.23 g/L were produced at 0.5 vvm and 1 vvm, respectively. Aeration rate of 0.5 vvm was used further study for high production of biomass and 2,3-BD.

#### Effect of pH

The acidity of fermentation is critical to the cell growth and metabolite production. In the case of 2,3-BD production, it was reported that the formation of neutral 2,3-BD could be a countermeasure against acidification [1]. It was found that the production of 2,3-BD was triggered by pH drop in *K. pneumoniae* G31 [32]. Forced pH fluctuation was implemented to increase the titer of 2,3-BD to 70 g/L in comparison with 52.5 g/L without pH control. In this work, we examined the effect of pH through cultivation ranging from pH 4 to pH 6 and without pH control. As illustrated in Fig. 6c, the production of 2,3-BD in X33-SD was not affected by the pH, reaching a final titer of about 10.8 g/L. The highest acetoin titer was observed at pH 6. The lowest glycerol concentration was detected at pH 6, reaching 0.46 g/L. At pH 4, cell growth was significantly retarded (data not shown). When pH was not controlled, pH value dropped from initial pH 6.8 to pH 5.2 at the end of the batch. In previous studies, pH was reported to have an impact on the activity of acetoin reductase. Through a two-stage pH control strategy, cell growth of *K. oxytoca* was favoured in the first stage where pH was uncontrolled and maintained at a set pH for 2,3-BD production [33]. The results obtained in this study indicate that *P. pastoris* could be a robust host for the production of 2,3-BD within a wide range of pH. We selected pH 5 for fed-batch cultivation, as optimal cell growth was achieved under this pH value.

Results of effect of cultivation conditions are summarized in Table 4. Comparison of results was performed using statistical analysis. The following conditions were selected for fed-batch cultivation: pH 5, 300 rpm and 0.5 vvm.

**Table 4:** Effect of cultivation conditions

	Agitation (rpm)			Aeration (vvm)			pH			
	300	400	500	0.1	0.5	1.0	4	5	6	Uncontrolled
OD_600_	23.65 ± 1.5^α^	34.8 ± 1.9^β^	42.35 ± 2.61^γ^	17.6 ± 0.8^δ^	22.95 ± 0.85^α^	25.1 ± 1^α^	17 ± 0.9^δ^	22.95 ± 1^α^	23 ± 1.2^α^	23.85 ± 1.3^α^
2,3-BD (g/L)	8.34 ± 0.62^α^	4.36 ± 32^β^	ND	11.07 ± 0.5^γ^	10.78 ± 0.3^γ^	8.87 ± 0.2^α^	10.82 ± 0.2^γ^	10.78 ± 0.6^γ^	10.97 ± 0.7^γ^	10.77 ± 0.9^γ^
Acetoin (g/L)	1.17 ± 0.02^α^	1.37 ± 0.02^β^	9.27 ± 0.81^γ^	0.97 ± 0.08^δ^	1.6 ± 0.12^ε^	1.89 ± 0.15^ε^	0.91 ± 0.04^α^	1.63 ± 0.1^γ^	2.75 ± 0.3^ζ^	0.99 ± 0.05^α^
Glycerol (g/L)	3.49 ± 0.1^α^	ND	ND	4.95 ± 0.3^β^	3.45 ± 0.2^α^	2.26 ± 0.2^γ^	4 ± 0.2^δ^	3.46 ± 0.18^α^	0.46 ± 0.03^ε^	4 ± 0.3^δ^
Ethanol (g/L)	0.66 ± 0.01^α^	ND	ND	2.43 ± 0.2^β^	0.46 ± 0.1^α^	ND	0.2 ± 0.01^γ^	0.47 ± 0.05^α^	0.01 ± 0^δ^	0.2 ± 0.01^γ^

Data indicate average values of duplicate experiments with standard deviation. Values with different Greek symbols between columns were significantly different (*P* < 0.05). ND indicates not detected

### Fed-batch cultivation in YPD

**Fig. 7:**
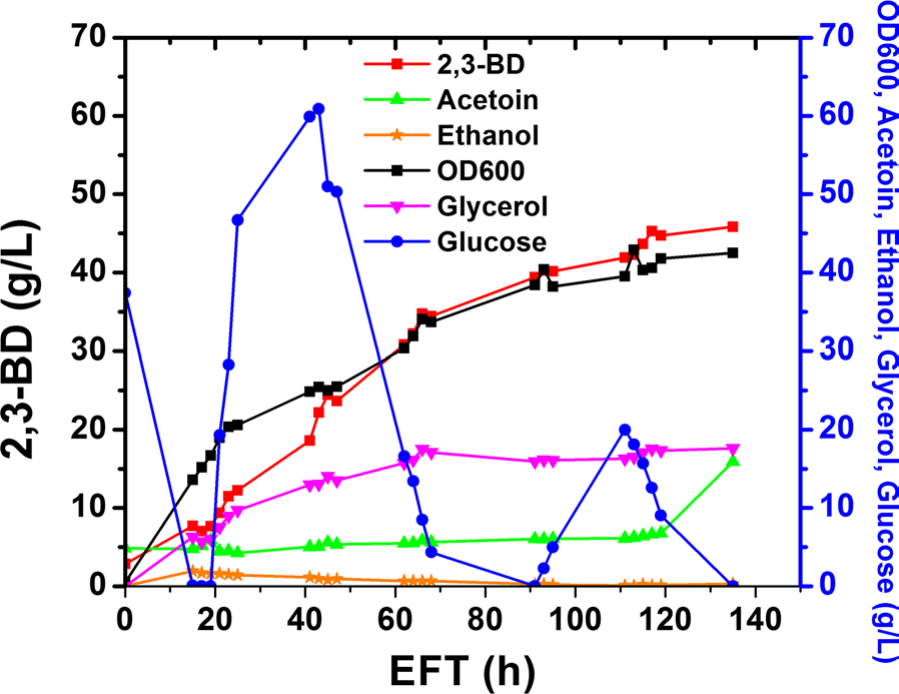
Time course of fed-batch cultivation. Fermentation was performed in 5-L Bioflo320 bioreactor with 2 L initial YPD media. 1 L of 500 g/L glucose was fed to bioreactor after glucose depletion

To scale up the production of 2,3-BD using strain X33-SD, fed-batch cultivation was performed. Batch cultivation was started in 2 L YPD media with initial glucose concentration of 40 g/L. The time course of fed-batch fermentation is shown in Fig. 7. Glucose was depleted at 19 h and feeding solution consisting of 500 g/L glucose was fed to the bioreactor. Fermentation lasted for 135 h with a final volume of approximately 2.5 L. Biomass and the titer of 2,3-BD steadily increased. The final OD_600_ reached 42.5 at the end of fed-batch. The production of 2,3-BD stagnated after 100 h and a final titer of 45.8 g/L was achieved. Ethanol was detected below 1 g/L. Final acetoin concentration was found to be 15.9 g/L. Glycerol production was 17.5 g/L at 66 h and remained unchanged at the end of cultivation. The yield of 2,3-BD on glucose of the whole fed-batch cultivation was 0.197 g/g, which is 39.4% of the theoretical yield. The productivity achieved in fed-batch fermentation was 0.34 g/L/h. Compared with shake flask cultivation, 2,3-BD yield obtained in fed-batch cultivation was relatively low. This could be due to the inhibitory effect of much higher concentration of glycerol and 2,3-BD in fed-batch cultivation. Moreover, YPD might not be a suitable medium for long-time fermentation. Medium optimization could be performed to boost the titer and yield of 2,3-BD.

### Statistical medium optimization

#### Effect of yeast extract

BSM is a defined medium commonly used for high cell density cultivation of *P. pastoris*. BSM consists of mineral salts without complex nitrogen source. To develop an appropriate medium for 2,3-BD production, we first investigated the effect of complex nitrogen source on the titer of 2,3-BD. Media containing 40 g/L glucose were used for the production of 2,3-BD. It was shown that only 0.5 ± 0.05 g/L 2,3-BD was detected in BSM, whilst 4.5 ± 0.7 g/L was obtained in BSM supplemented with 10 g/L yeast extract (Additional file 1: Figure S1). It has been previously reported that the addition of complex nitrogen source such as yeast extract and corn steep liquor was beneficial to boost the titer of 2,3-BD [33, 34]. Therefore, it is reasonable to factor in yeast extract for optimization of BSM in this work.

#### Plackett–Burman and Box–Behnken design

Plackett–Burman design was used to screen significant factors affecting the production of 2,3-BD. Twelve-run shake flask cultivation was carried out based on the matrix (Additional file 4: Tables S1–S2) generated by Minitab 15. The responses showed good fit to the first-order model (Additional file 4: Equation S1). The *F* value for the fitted equation is 6.93 with *P* = 0.04, indicating the good fitness of the model. Statistical analysis (Additional file 4: Table S3 and Additional file 2: Figure S2) revealed that significance of medium components impacting the titer of 2,3-BD in the following order: KH_2_PO_4_ > yeast extract > MgSO_4_ > PTM1 > K_2_SO_4_ > (NH_4_)_2_SO_4_ > CaSO_4_. Apparently, KH_2_PO_4_, yeast extract and MgSO_4_ were the key variables and were chosen to determine their optimal levels using Box–Behnken design (Additional file 4: Tables S4–S7). The responses showed good fit to the quadratic model. It was found that the highest titer of 2,3-BD was achieved when the concentration of yeast extract, KH_2_PO_4_ and MgSO_4_ was 15, 21.5 and 2.85 g/L, respectively.

#### Fed-batch cultivation using optimized BSMY media

**Fig. 8:**
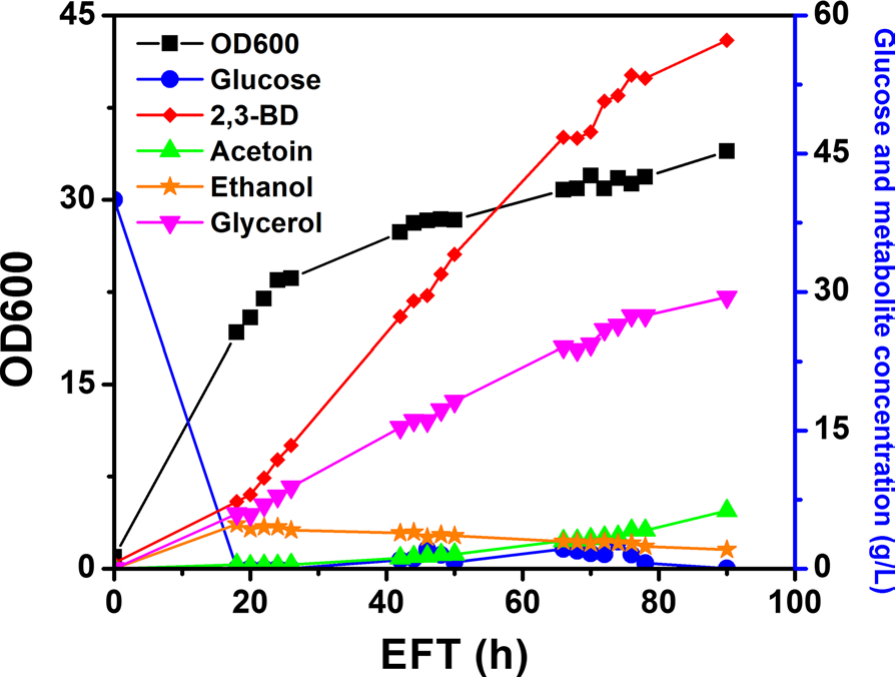
Time course of fed-batch fermentation using optimized BSMY medium. Bioreactor contained 2 L of initial media. 40 g/L glucose was used as initial substrate and 1 L of 500 g/L glucose solution was used as feeding solution in fed-batch

**Fig. 9:**
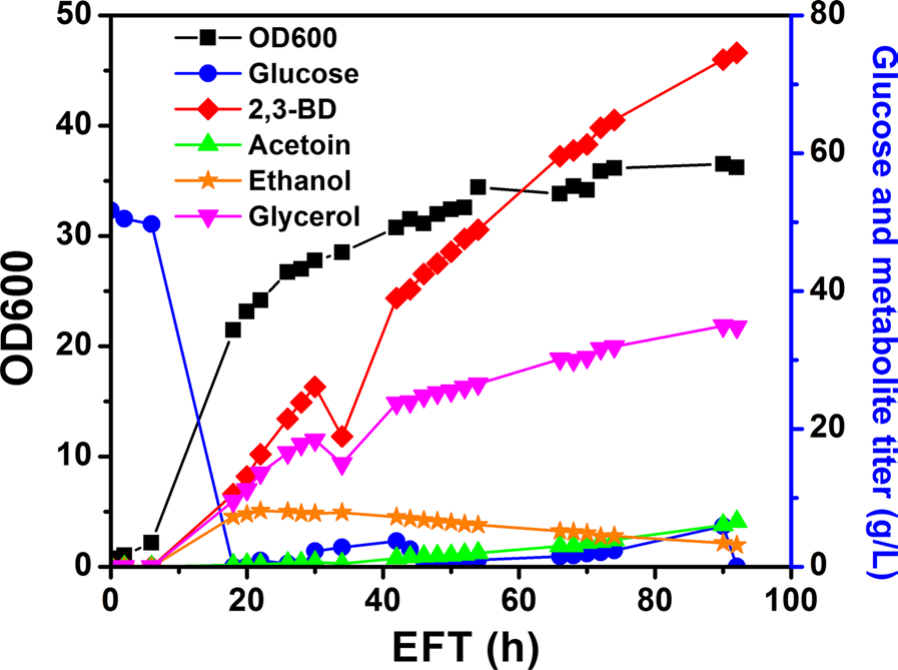
Time course of fed-batch fermentation using optimized BSMY medium. Bioreactor contained 2 L of initial media. 60 g/L glucose was used as initial substrate and 1 L of 500 g/L glucose solution was used as feeding solution in fed-batch

The optimized BSMY media consist of 15 g/L yeast extract, 21.5 g/L KH_2_PO_4_ and 2.85 g/L MgSO_4_. Other medium components were kept the same as the standard BSM. Three fed-batch cultivations were performed to evaluate the performance of the optimized medium. Upon the depletion of initial glucose in the batch within about 18 h, feeding solution containing 250 or 500 g/L glucose was fed to the bioreactor. Glucose feed rate was adjusted to maintain a low glucose concentration. In fed-batch cultivation with 40 g/L initial glucose concentration and 1 L of 250 g/L glucose feeding solution, the fermentation was completed within 49 h (Additional file 3: Figure S3). The final titer of 2,3-BD was 41 g/L with a yield on glucose of 0.31 g/g. To boost the titer, higher concentrations of initial glucose and feeding glucose were studied. As can be seen from Figs. 8 and 9, fed-batch cultivation was completed within 90 h, which was significantly shortened in comparison with YPD (135 h). The highest titer of 2,3-BD was achieved when 60 g/L of initial glucose and 1 L of 500 g/L glucose feeding solution were used, reaching 74.5 g/L. In contrast, the highest titer obtained in YPD was 45 g/L in fed-batch fermentation with a much longer cultivation time. Comparison of the fed-batch cultivation results are summarized in Additional file 4: Table S8. Compared with YPD media, optimized BSMY could support faster glucose consumption without accumulating excessive glucose in the culture. Glycerol was the major by-product, reaching about 35 g/L in the final culture. Ethanol and acetoin were detected at low titer.

## Discussion

Biological production of bulk chemicals has garnered increasing attention in recent years. Production of optically pure 2,3-BD has been extensively investigated due to the wide applications of this platform chemical. Selection of a suitable host for metabolic engineering is crucial to the economic feasibility of the process. Engineering of native producers such as *K. pneumoniae* [47], *S. marcescens* [48] and *B. licheniformis* [49] requires the deletion of 2,3-BDH. An alternative strategy to produce pure optical isomer of 2,3-BD was to engineer heterologous hosts such as *E. coli* [10], *S. cerevisiae* [14] and *L. lactis* [25]. To boost the titer of 2,3-BD, metabolic engineering and process engineering strategies could be employed. Metabolic engineering strategies include overexpression of the 2,3-BD synthesis pathway genes, inactivation of the competing pathway genes and co-factor engineering. Overexpression of *budA* and *budB* in a *K. pneumoniae* strain resulted in 60% increase of 2,3-BD compared with parental strain [50]. Minimization of by-products was necessary to increase the yield of 2,3-BD. Lactic acid was formed as by-product in *K. pneumoniae*, *K. oxytoca* [51] and *E. cloacae* [37], and deletion of *ldhA* (encoding lactate dehydrogenase) enhanced the production of 2,3-BD in those strains.

Co-factor imbalance is a major bottleneck for the production of 2,3-BD from glucose. Two moles of NADH were generated during the glycolysis of 1 mol of glucose. Only 1 mol of NADH was consumed to reduce acetoin to 2,3-BD, causing a surplus of NADH. NAD^+^ can be recycled through NADH consumption processes such as the formation of lactic acid, ethanol and glycerol. As was mentioned previously, glycerol was produced as the major by-product in engineered *P. pastoris* strains, similar to that observed in *S. cerevisiae* [11, 12]. In *S. cerevisiae*, NADH reoxidation could be achieved through several mechanisms such as alcoholic fermentation, glycerol formation and respiration [52]. Under anaerobic conditions where respiration cannot occur, glycerol formation was employed by *P. pastoris* as a major redox sink to recycle NAD^+^, as *P. pastoris* is a Crabtree effect-negative yeast and produces much less ethanol than *S. cerevisiae*. Similar glycerol formation mechanism may also exist in engineered *Z. mobilis* [46]. More glycerol was produced when more 2,3-BD was formed. In fed-batch cultivation, 35 g/L glycerol was observed with 74.5 g/L 2,3-BD (Fig. 9). In contrast, no glycerol was detected when acetoin was the major product and no 2,3-BD was formed at agitation speed of 500 rpm (Fig. 6). In *S. cerevisiae*, glycerol is formed through the two GPDs. Disruption of the two GPDs in *S. cerevisiae* almost abolished glycerol formation and enhanced 2,3-BD production [29]. Similar strategy could be implemented in *P. pastoris* to minimize glycerol synthesis and improve 2,3-BD titer. Co-factor engineering is an efficient strategy to balance the NAD^+^/NADH ratio. Expression of NADH oxidase gene *L. lactis noxE* in engineered *S. cerevisiae* strains lacking pyruvate decarboxylase resulted in increased 2,3-BD yield and productivity [29, 35, 53]. Overexpression of *E. coli udhA* gene (encoding a transhydrogenase) also led to enhanced production of 2,3-BD in engineered *B. subtilis* [39]. However, expression of *E. coli udhA* was found not beneficial to boost the titer of 2,3-BD in our study.

Cultivation conditions such as pH and DO should be optimized to achieve optimal production of 2,3-BD. Cultivation pH can affect the production of 2,3-BD as well as cell growth and thus should be investigated. It was found that metabolite profile was highly associated with the cultivation pH in *Klebsiella* sp. Zmd30 and optimal existed for high production of 2,3-BD [54]. Biomass accumulation and 2,3-BD production may favour different pH. Two-stage pH control scheme by controlling pH at 7.5 for cell growth and then reduced to 6.5 for 2,3-BD synthesis has been successfully implemented in *E. cloacae* [55]. In this work, variation of pH between 4 and 6 did not show a significant difference of 2,3-BD titer, albeit cell growth was retarded at pH 4. DO is critical for achieving high titer of 2,3-BD. Complicated DO control schemes such as volumetric oxygen transfer coefficient *K*_L_*a* [34] and respiratory quotient [56] have been reported. Since DO is governed by agitation speed and aeration rate, it would be easier to control DO through optimizing those two parameters. Chan et al. [57] implemented a “one variable a time” strategy to optimize pH, agitation speed, aeration rate and maltodextrin concentration for the production of 2,3-BD in *K. oxytoca*. Three-stage agitation speed increased the titer of 2,3-BD by 9.8% in *B. amyloliquefaciens* [58]. In our work, product profile was related to agitation speed and it could be manipulated to achieve production of acetoin or 2,3-BD.

YPD is a commonly used rich medium for yeast cultivation. However, it is not viable to use such expensive medium for industrial-scale production due to the costly yeast extract and peptone. Moreover, glucose accumulation was observed during long-time fermentation (Fig. 9). The slow utilization of glucose was also observed in the fermentation of engineered *S. cerevisiae* strains using YPD where cultivation time was over 300 h [12, 14]. Statistical medium optimization is an efficient approach to develop a suitable medium for enhanced production of 2,3-BD. It has been successfully used to develop industrial medium for other 2,3-BD producers [59, 60]. Despite minimal medium should be employed for industrial fermentation to reduce cost, the addition of complex nitrogen source such as yeast extract, casamino acids and corn steep liquor has been reported to increase the titer and yield of 2,3-BD in *K. oxytoca* [33], *P. polymyxa* [34], *Raoultella ornithinolytica* B6 [61] and *B. subtilis* [62]. Cultivation of *P. pastoris* strains in minimal salt medium led to very low titer of 2,3-BD. Therefore, yeast extract was used for the optimization of BSM to avoid compromising the titer of 2,3-BD. The three significant variables were identified through statistical analysis. KH_2_PO_4_ was found to have a negative impact on the titer of 2,3-BD and should be reduced. This is consistent with a previous report optimizing BSM for enhanced phytase production in *P. pastoris* [16]. The optimized medium showed more robust performance than YPD in terms of higher yield, shorter fermentation time, faster glucose consumption and higher 2,3-BD titer.

Yeast extract was still used in the optimized medium due to its role in enhancing 2,3-BD titer. Feasibility of using other inexpensive nitrogen source such as corn steep liquor [63, 64] to partially substitute yeast extract could be explored with similar medium optimization strategy described in this work.

A comparison of the production of 2,3-BD using various strains is summarized in Table 5. Efficient producers of 2,3-BD such as *K. pneumoniae* and *K. oxytoca* are predominantly native hosts. The highest titer of 2,3-BD in bacteria was reported in *K. pneumoniae* SDM, reaching 150 g/L with a productivity of 4.21 g/L/h [43]. The highest production yield of 2,3-BD on glucose was 0.49 g/g achieved in *K. oxytoca* [42], close to the theoretical yield of 0.5 g/g. The yield also reached 0.487 g/g in an engineered *B. subtilis* [39]. The best yield in a heterologous host was reported in an engineered *E. coli*, reaching 0.49 g/g in shake flask cultivation with a titer of 54 g/L [65]. Engineered *S. cerevisiae* has achieved success in the production of 2,3-BD with the best reported titer being 154 g/L [35]. However, the yield obtained in *S. cerevisiae* was relatively low, mostly between 0.2 and 0.4 g/g. In this work, engineered *P. pastoris* strain was able to achieve 0.3 g/g production yield, comparable to that of *S. cerevisiae*. Further metabolic engineering should be performed to increase the yield and productivity thereby enhancing the economic viability.

**Table 5:** Comparison of 2,3-BD production in various microorganisms

Species	Titer (g/L)	Operation strategy	Enantiopurity (%)	Yield (g/g)	Feed stock	2,3-BD productivity (g/L/h)	References
*S. cerevisiae*	43.6	Fed-batch	97	0.27	Xylose	0.15	[12]
*S. cerevisiae*	96.2	Fed-batch	NA	0.28	Glucose	0.39	[11]
*S. cerevisiae*	154.3	Fed-batch	NA	0.404	Glucose	1.97	[35]
*S. cerevisiae*	100	Fed-batch	98	0.35	Glucose and galactose	0.33	[14]
*E. coli*	6.9	Shake flask	99	0.31	Glucose	0.14	[36]
*E. coli*	73.8	Fed-batch	NA	0.41	Glucose	1.19	[30]
*E. coli*	115	Fed-batch	99	0.42	Glucose	1.44	[9]
*E. aerogenes*	152	Fed-batch	97.5	0.489	Glucose and xylose	3.5	[37]
*E. aerogenes*	140	Fed-batch	NA	NA	Sugarcane molasses	2.59	[38]
*B. subtilis*	49.29	Shake flask	99	0.47	Glucose	0.224	[4]
*B. subtilis*	130.7	Fed-batch	99	0.487	Glucose	0.459	[39]
*B. licheniformis*	123.7	Fed-batch	99	NA	Glucose	2.95	[6]
*B. amyloliquefaciens*	132.9	Fed-batch	NA	0.45	Glucose	2.95	[40]
*K. oxytoca*	131.5	Fed-batch	NA	0.44	Crude glycerol	0.84	[41]
*K. oxytoca*	117.4	Fed-batch	NA	0.49	Glucose	1.2	[42]
*K. pneumoniae*	150	Fed-batch	NA	NA	Glucose	4.21	[43]
*S. marcescens*	152	Fed-batch	NA	0.463	Sucrose	2.67	[44]
*P. polymyxa*	36.92	Batch	98		Raw inulin extract	0.88	[8]
*C. glutamicum*	6.3	Batch	NA	0.33	Glucose	0.2	[45]
*Z. mobilis*	15	Batch	NA	0.16	Glucose, xylose	0.3	[46]
*P. pastoris*	75	Fed-batch	99	0.3	Glucose	0.81	This study

Construction of engineered hosts generally requires antibiotic resistance as selection marker. The use of antibiotic-resistant microorganisms may not be ideal for industrial applications due to possible environmental impact. This concern could be addressed using nutritional auxotrophy as selection marker such as histidine-deficiency (*his4*) in *P. pastoris*. The antibiotic resistance gene could also be removed through proper molecular techniques such as flippase-mediated homologous recombination [66].

Compared with other engineered heterologous systems depending on plasmid-based expression of 2,3-BD synthesis genes, gene expression in *P. pastoris* was based on genome-targeting cassette and eliminated the need of episomal plasmids. Combined with the high growth rate, high alcohol tolerance and GRAS status, engineered *P. pastoris* could become a robust host for the production of 2,3-BD.

## Conclusions

*Pichia pastoris* was engineered into a microbial cell factory to produce bulk chemical (2R, 3R)-2,3-BD using glucose as feedstock for the first time. The endogenous 2,3-BDH could support efficient conversion of acetoin to 2,3-BD. Statistical medium optimization was a useful tool to boost the titer of 2,3-BD. The highest titer of (2R, 3R)-2,3-BD reached 74.5 g/L in a fed-batch cultivation using optimized medium. *P. pastoris* was proved a versatile platform for biofuel production other than heterologous protein production. The engineered *P. pastoris* strain could be a good starting point for further metabolic and process engineering to achieve a novel host for cost-effective production of (2R, 3R)-2,3-BD.

## Additional files

**Additional file 1: Figure S1.** Effect of yeast extract on 2,3-BD titer. 100 mL media containing 40 g/L glucose were used to cultivate strain X33-SD. Error bar indicate standard deviation of three replicate experiments.

**Additional file 2: Figure S2.** Pareto graph of the seven variables.

**Additional file 3: Figure S3.** Time course of fed-batch fermentation using optimized BSMY medium. Bioreactor contained 2 L of initial media. 40 g/L glucose as initial substrate and 1 L of 250 g/L glucose solution as feeding solution.

**Additional file 4.** Supplementing tables in this work. **Table S1.** Level code for variables based on Plackett-Burman design. **Table S2.** Cultivation results of Plackett-Burman design. **Table S3.** Analysis results of Plackett-Burman design. **Table S4.** Design of Box-Behnken experiments. **Table S5.** Concentration level for Box-Behnken design. **Table S6.** Cultivation results of Box-Behnke design. **Table S7.** Analysis results of Box-Behnken design. **Table S8.** Comparison of fed-batch cultivations.
