# Supplementary figures and images for: Production of (2R, 3R)-2,3-butanediol using engineered Pichia pastoris: strain construction, characterization and fermentation
Source: Biotechnol Biofuels. 2018 Feb 12;11:35. doi: 10.1186/s13068-018-1031-1 (PMC5808657; doi:10.1186/s13068-018-1031-1)

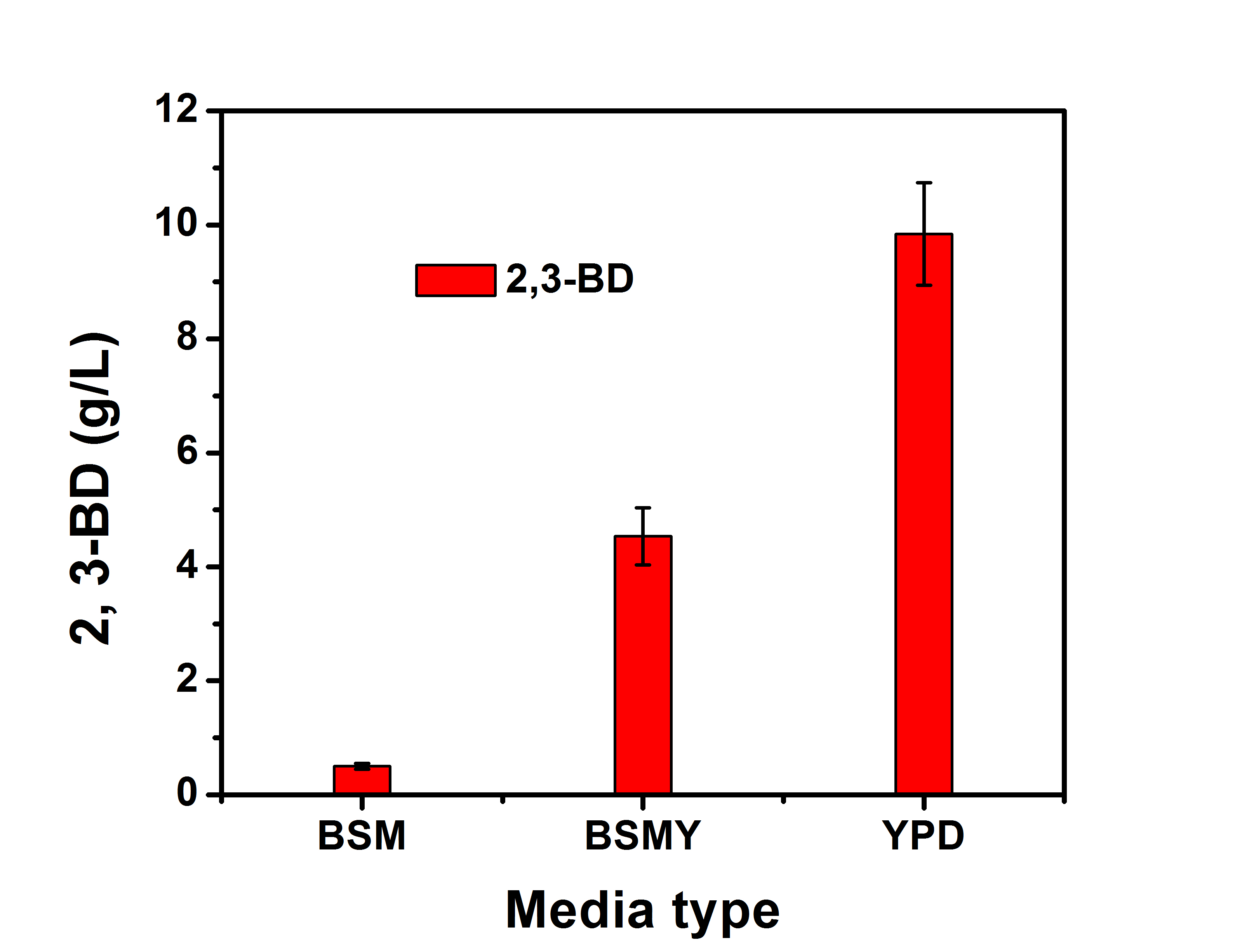

Supplement: Supplementary file 1 — Additional file 1: Figure S1. Effect of yeast extract on 2,3-BD titer. 100 mL media containing 40 g/L glucose were used to cultivate strain X33-SD. Error bar indicate standard deviation of three replicate experiments. [file 13068_2018_1031_MOESM1_ESM.jpg]

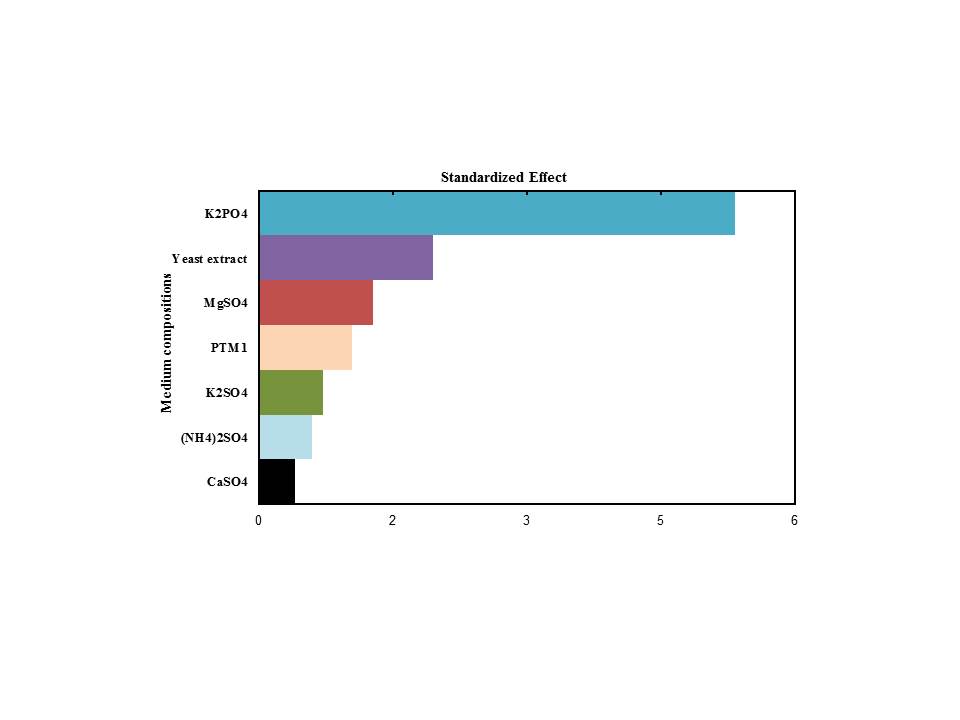

Supplement: Supplementary file 2 — Additional file 2: Figure S2. Pareto graph of the seven variables. [file 13068_2018_1031_MOESM2_ESM.jpg]

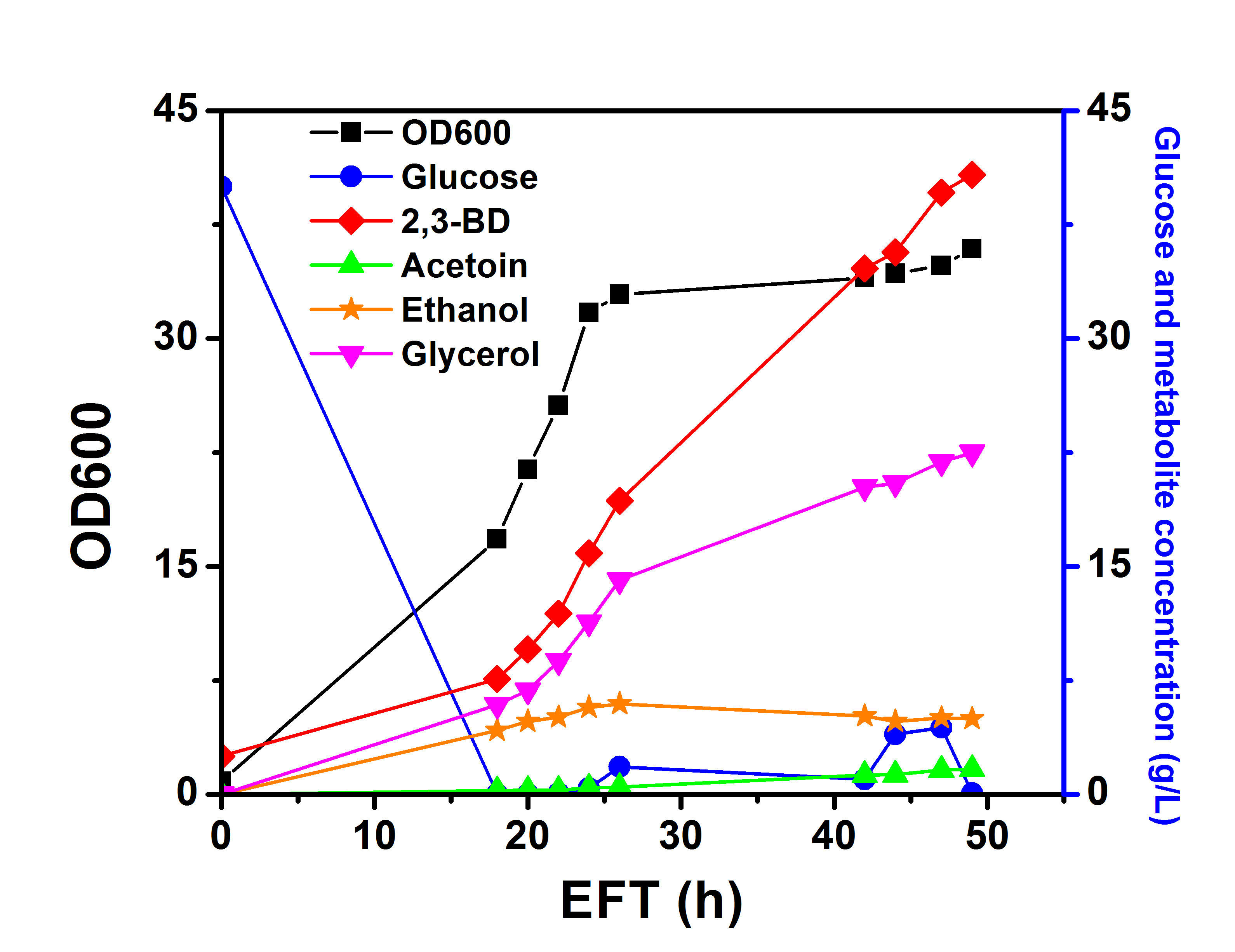

Supplement: Supplementary file 3 — Additional file 3: Figure S3. Time course of fed-batch fermentation using optimized BSMY medium. Bioreactor contained 2 L of initial media. 40 g/L glucose as initial substrate and 1 L of 250 g/L glucose solution as feeding solution. [file 13068_2018_1031_MOESM3_ESM.jpg]
