# Supplementary material for: Production of (2R, 3R)-2,3-butanediol using engineered Pichia pastoris: strain construction, characterization and fermentation
Source: Biotechnol Biofuels. 2018 Feb 12;11:35. doi: 10.1186/s13068-018-1031-1 (PMC5808657; doi:10.1186/s13068-018-1031-1)
Supplement: Supplementary file 4 — Additional file 4. Supplementing tables in this work. Table S1. Level code for variables based on Plackett-Burman design. Table S2. Cultivation results of Plackett-Burman design. Table S3. Analysis results of Plackett-Burman design. Table S4. Design of Box-Behnken experiments. Table S5. Concentration level for Box-Behnken design. Table S6. Cultivation results of Box-Behnke design. Table S7. Analysis results of Box-Behnken design. Table S8. Comparison of fed-batch cultivations. [file 13068_2018_1031_MOESM4_ESM.docx]

**Table S1** Level code for variables based on Plackett-Burman design

| Variable  Run | Yeast extract  X1 | CaSO_4_  X2 | KH_2_PO_4_  X3 | K_2_SO_4_  X4 | MgSO_4_  X5 | (NH4)_2_SO_4_  X6 | PTM1  X7 |
| --- | --- | --- | --- | --- | --- | --- | --- |
| 1 | 1 | -1 | 1 | -1 | -1 | -1 | 1 |
| 2 | 1 | 1 | -1 | -1 | -1 | 1 | -1 |
| 3 | -1 | 1 | 1 | 1 | -1 | -1 | -1 |
| 4 | 1 | -1 | 1 | -1 | 1 | 1 | -1 |
| 5 | 1 | 1 | -1 | 1 | -1 | 1 | 1 |
| 6 | 1 | 1 | 1 | 1 | 1 | -1 | -1 |
| 7 | -1 | 1 | 1 | -1 | 1 | 1 | 1 |
| 8 | -1 | -1 | 1 | 1 | -1 | 1 | 1 |
| 9 | -1 | -1 | -1 | 1 | 1 | 1 | -1 |
| 10 | 1 | -1 | -1 | 1 | 1 | -1 | 1 |
| 11 | -1 | 1 | -1 | -1 | 1 | -1 | 1 |
| 12 | -1 | -1 | -1 | -1 | -1 | -1 | -1 |

**Table S2.** Cultivation results of Plackett-Burman design

| Run | 1 | 2 | 3 | 4 | 5 | 6 | 7 | 8 | 9 | 10 | 11 | 12 |
| --- | --- | --- | --- | --- | --- | --- | --- | --- | --- | --- | --- | --- |
| 2,3-BD (g/L) | 5.066 | 7.545 | 3.242 | 3.444 | 6.280 | 2.998 | 3.474 | 2.685 | 4.935 | 7.029 | 5.415 | 5.490 |

**Table S3** Analysis results of Plackett-Burman design

| Code | Variables | Low level (g/L) (-1) | High level (g/L) (+1) | Effects | Standard error | T value | P > \|T\| |
| --- | --- | --- | --- | --- | --- | --- | --- |
| X1 | Yeast extract | 5 | 15 | 1.187 | 0.2159 | 2.75 | 0.051 |
| X2 | CaSO_4_ | 0.3 | 0.9 | 0.051 | 0.2159 | 0.12 | 0.912 |
| X3 | KH_2_PO_4_ | 21.45 | 64.35 | -2.631 | 0.2159 | -6.09 | 0.004 |
| X4 | K_2_SO_4_ | 7.165 | 21.495 | -0.544 | 0.2159 | -1.26 | 0.276 |
| X5 | MgSO_4_ | 2.855 | 8.565 | -0.502 | 0.2159 | -1.16 | 0.309 |
| X6 | (NH4)_2_SO_4_ | 2.585 | 7.755 | -0.146 | 0.2159 | -0.34 | 0.752 |
| X7 | PTM1 | 2 ml/L | 6 ml/L | 0.383 | 0.2159 | 0.89 | 0.426 |

S = 0.747792 PRESS = 20.1310

R^2^ = 92.39% R^2^ (ADJ) = 79.06%

First-order model:

Y = *β0 + Σβi*X*i* (*i* = 1,..., 7) (equation S1)

Fitted model: Y=4.800+1.187X_1_+0.051X_2_-2.631X_3_-0.544X_4_-0.502X_5_-0.146X_6_+0.383X_7_

**Table S4** Design of Box-Behnken experiments

| Number  Variable | 1 | 2 | 3 | 4 | 5 | 6 | 7 | 8 | 9 | 10 | 11 | 12 | 13 | 14 | 15 |
| --- | --- | --- | --- | --- | --- | --- | --- | --- | --- | --- | --- | --- | --- | --- | --- |
| Yeast extract | -1 | -1 | 1 | 1 | 0 | 0 | 0 | 0 | -1 | 1 | -1 | 1 | 0 | 0 | 0 |
| KH_2_PO_4_ | -1 | 1 | -1 | 1 | -1 | 1 | -1 | 1 | 0 | 0 | 0 | 0 | 0 | 0 | 0 |
| MgSO_4_ | 0 | 0 | 0 | 0 | -1 | -1 | 1 | 1 | -1 | -1 | 1 | 1 | 0 | 0 | 0 |

**Table S5** Concentration level for Box-Behnken design

| Factors | Level (g/L) | | |
| --- | --- | --- | --- |
|  | -1 | 0 | +1 |
| KH_2_PO_4_ | 21.45 | 42.9 | 64.35 |
| Yeast extract | 5 | 10 | 15 |
| MgSO_4_ | 2.855 | 5.7 | 8.565 |

**Table S6** Cultivation results of Box-Behnke design

| Number | 1 | 2 | 3 | 4 | 5 | 6 | 7 | 8 | 9 | 10 | 11 | 12 | 13 | 14 | 15 |
| --- | --- | --- | --- | --- | --- | --- | --- | --- | --- | --- | --- | --- | --- | --- | --- |
| 2,3-BD (g/L) | 4.09 | 4.66 | 7.90 | 5.18 | 8.73 | 5.51 | 8.37 | 5.64 | 5.31 | 7.93 | 5.55 | 7.04 | 7.58 | 7.48 | 7.41 |

**Table S7** Analysis results of Box-Behnken design

| Factors | Coefficient | Standard error | T | P |
| --- | --- | --- | --- | --- |
| Constant | 7.4900 | 0.3415 | 21.930 | 0.000 |
| KH_2_PO_4_ | -1.0250 | 0.2091 | -4.901 | 0.004 |
| Yeast extract | 1.0425 | 0.2091 | 4.985 | 0.004 |
| MgSO_4_ | -0.1100 | 0.2091 | -0.526 | 0.621 |
| KH_2_PO_4_ * KH_2_PO_4_ | -0.7013 | 0.3079 | -4.243 | 0.008 |
| Yeast extract * Yeast extract | -1.3062 | 0.3079 | -2.278 | 0.072 |
| MgSO_4_ * MgSO_4_ | 0.2737 | 0.3079 | 0.889 | 0.415 |
| KH_2_PO_4_ * Yeast extract | -0.7975 | 0.2958 | -2.696 | 0.043 |
| KH_2_PO_4_ * MgSO_4_ | 0.1225 | 0.2958 | -0.955 | 0.383 |
| Yeast extract * MgSO_4_ | -0.2825 | 0.2958 | 0.414 | 0.696 |

S = 0.591557 PRESS = 27.7945

R^2^ = 94.20% R^2^ (ADJ) = 83.76%

Quadratic model:

Y_i_ = b_0_ + b_1_X_1_ + b_2_X_2_ + b_3_X_3_ + b_12_X_1_X_2_ + b_13_X_1_X_3_ + b_23_X_2_X_3_ + b_11_X_2_^2^ + b_22_X_2_^2^ + b_33_X_3_^2^ (equation S2)

Fitted model:

Y = 7.4900 + 1.0425*X*_1_ - 1.0250X_2_ - 0.1100X_3_ - 0.7975X_1_X_2_ - 0.2825X_1_X_3_ + 0.1225X_2_X_3_ – 1.30625X_1_^2^ – 0.70125X_2_^2^ + 0.27375X_3_^2^

**Table S8** Comparison of fed-batch cultivations

| Cultivation media | Duration (h) | 2,3–BD (g/L) | Productivity (g/L/h) | Yield (g/g) |
| --- | --- | --- | --- | --- |
| YPD | 135 | 45 | 0.33 | 0.194 |
| BSMY | 49 | 41 | 0.84 | 0.31 |
| BSMY | 90 | 57.3 | 0.64 | 0.246 |
| BSMY | 92 | 74.5 | 0.81 | 0.3 |

Theoretical yield is 0.5 g/g.
